# 2-Amino-*N*-(2-hydr­oxy-3-methoxy­benzyl­idene)aniline

**DOI:** 10.1107/S1600536808016292

**Published:** 2008-06-07

**Authors:** Mohammed H. Al-Douh, Shafida A. Hamid, Hasnah Osman, Reza Kia, Hoong-Kun Fun

**Affiliations:** a School of Chemical Sciences, Universiti Sains Malaysia, 11800 USM, Penang, Malaysia; b X-ray Crystallography Unit, School of Physics, Universiti Sains Malaysia, 11800 USM, Penang, Malaysia

## Abstract

In the title compound, C_14_H_14_N_2_O_2_, the dihedral angle between the two benzene rings is 9.67 (10)°. Two intra­molecular O—H⋯N and N—H⋯N hydrogen bonds involving the hydr­oxy and amino groups generate *S*(6) and *S*(5) ring motifs, respectively. In the crystal structure, N—H⋯O hydrogen bonds link neighboring mol­ecules. Mol­ecules are also stacked in a head-to-tail fashion along the *c* axis through π–π inter­actions [centroid–centroid separation of 3.7357 (12) Å] and are further linked by weak inter­molecular C—H⋯π inter­actions, giving a zigzag arrangement along the *b* axis.

## Related literature

For related literature on hydrogen bond motifs, see: Bernstein *et al.* (1995 ▶). For values of bond lengths, see: Allen *et al.* (1987 ▶). For the biological activity of imines, see, for example: Singh & Dash (1988 ▶); More *et al.* (2001 ▶); Baseer *et al.* (2000 ▶); El-Masry *et al.* (2000 ▶); Kabeer *et al.* (2001 ▶); Kuz’min *et al.* (2000 ▶); Desai *et al.* (2001 ▶). For related structures, see, for example: Corden *et al.* (1996 ▶); Govindasamy *et al.* (1999 ▶). For synthesis, see: Al-Douh *et al.* (2006 ▶, 2007 ▶). For related literature, see: Berger (2001 ▶); Elerman & Kabak (1997 ▶); Latif *et al.* (1983 ▶); Liu *et al.* (2006 ▶); Shah *et al.* (2008 ▶).
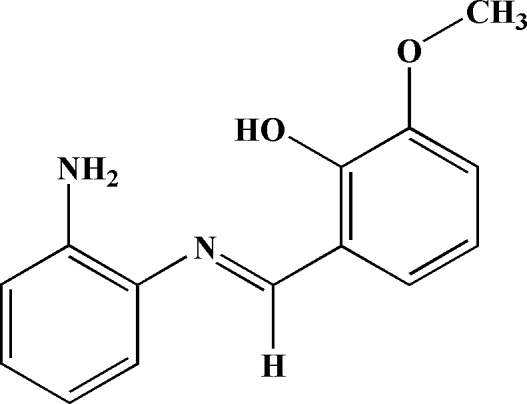


## Experimental

### 

#### Crystal data

C_14_H_14_N_2_O_2_
*M* *_r_* = 242.27
Monoclinic, 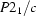

*a* = 13.2790 (6) Å
*b* = 14.4810 (6) Å
*c* = 6.1928 (3) Å
β = 103.116 (3)°
*V* = 1159.77 (9) Å^3^
*Z* = 4
Mo *K*α radiation
μ = 0.09 mm^−1^
*T* = 100.0 (1) K
0.45 × 0.15 × 0.05 mm

#### Data collection

Bruker SMART APEXII CCD area-detector diffractometer
Absorption correction: multi-scan (*SADABS*; Bruker, 2005 ▶) *T*_min_ = 0.959, *T*_max_ = 0.996
19968 measured reflections
3402 independent reflections
2532 reflections with *I* > 2σ(*I*)
*R*_int_ = 0.052

#### Refinement

*R*[*F* ^2^ > 2σ(*F* ^2^)] = 0.072
*wR*(*F* ^2^) = 0.206
*S* = 1.06
3402 reflections
194 parameters
H atoms treated by a mixture of independent and constrained refinement
Δρ_max_ = 0.66 e Å^−3^
Δρ_min_ = −0.31 e Å^−3^

### 

Data collection: *APEX2* (Bruker, 2005 ▶); cell refinement: *APEX2*; data reduction: *SAINT* (Bruker, 2005 ▶); program(s) used to solve structure: *SHELXTL* (Sheldrick, 2008 ▶); program(s) used to refine structure: *SHELXTL*; molecular graphics: *SHELXTL*; software used to prepare material for publication: *SHELXTL* and *PLATON* (Spek, 2003 ▶).

## Supplementary Material

Crystal structure: contains datablocks global, I. DOI: 10.1107/S1600536808016292/sj2510sup1.cif

Structure factors: contains datablocks I. DOI: 10.1107/S1600536808016292/sj2510Isup2.hkl

Additional supplementary materials:  crystallographic information; 3D view; checkCIF report

## Figures and Tables

**Table 1:** Hydrogen-bond geometry (Å, °) *Cg*1 and *Cg*2 are centroids of the C1–C6 and C8–C13 rings, respectively.

*D*—H⋯*A*	*D*—H	H⋯*A*	*D*⋯*A*	*D*—H⋯*A*
O1—H1O1⋯N1	0.88	1.77	2.602 (2)	158
N2—H1N2⋯O1^i^	0.93 (3)	2.56 (3)	3.030 (3)	111 (2)
N2—H1N2⋯O2^i^	0.93 (3)	2.29 (3)	3.181 (3)	161 (3)
N2—H2N2⋯N1	0.87 (3)	2.23 (3)	2.759 (3)	119 (2)
C3—H3⋯*Cg*1^ii^	0.94 (3)	2.64 (3)	3.458 (2)	145 (2)
C11—H11⋯*Cg*2^iii^	0.91 (3)	2.87 (3)	3.538 (2)	142 (2)

Symmetry codes: (i) 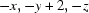; (ii) 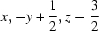; (iii) 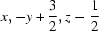.

**Fig. 1.**
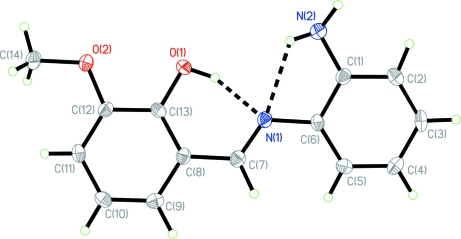
The molecular structure of the title compound, showing 50% probability displacement ellipsoids and the atomic numbering. Intramolecular H bonds are drawn as dashed lines.

**Fig. 2.**
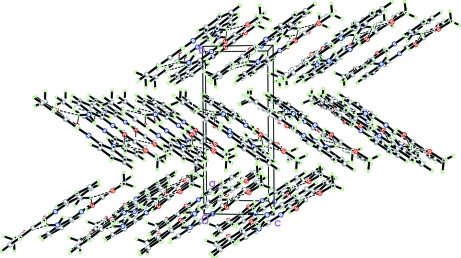
The crystal packing of (I) showing the zigzag stacking arrangement along the b axis. H bonds are drawn as dashed lines.

**Fig. 3.**
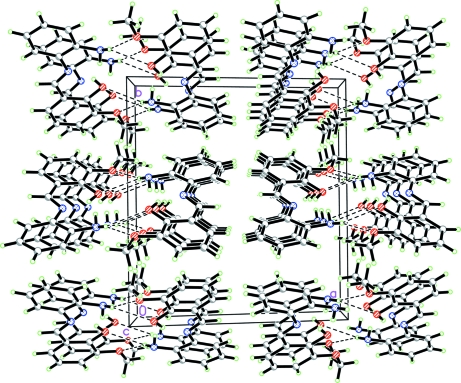
The crystal packing of (I), viewed along the c axis showing the molecular stacking. H bonds are drawn as dashed lines.

## supplementary crystallographic information

### Comment

Imines are an important class of compounds and rank among the most versatile synthetic organic intermediates, which are important for the synthesis of biologically important compounds (Singh & Dash, 1988; More *et al.*, 2001; Baseer *et al.*, 2000; El-Masry *et al.*, 2000; Kabeer *et al.,* 2001; Kuz'min *et al.*, 2000; Desai *et al.*, 2001). Berger (2001) evaluated some *bis*-Schiff bases using 1138 and Sc-7 yeast assays, and a A2780 cytotoxicity test. They showed significant activity in a single dose test. The reactions of some phenolic aldehydes with *o*-phenylenediamine have been examined in some detail including the isolation of the title compound (Latif *et al.*, 1983). Our group has been actively involved in synthesizing *bis*-Schiff bases and investigating their DNA binding ability using spectroscopic techniques employing calf thymus DNA (Shah *et al.*, 2008). We have also obtained single crystals of benzimidazole and the *bis*-Schiff base derived from the title compound (I) and their structures are consistent with those reported earlier (Elerman & Kabak, 1997; Liu *et al.*, 2006). However, the crystal structure of compound (I) was never reported and we present its structure here (Fig. 1).

The C1–C6 benzene ring is not coplanar with the phenylenediamine and makes a dihedral angle of 9.67 (10)° with C8–C13 benzene ring. Two intramolecular O1—H1O1···N1 and N2—H2N2···N1 hydrogen bonds generate S(6) and S(5) ring motifs, respectively (Bernstein *et al.*, 1995). Bond lengths and angles in the title compound have normal values (Allen *et al.*, 1987). The crystal is stabilized by intramolecular O—H···N and N—H···N and intermolecular N—H···O hydrogen bonds. Molecules (Fig. 2) are also arranged into zig-zag chains by C—H···π interactions along the *b*-axis (Table 1); *Cg1* and *Cg2* are the centroids of the C1–C6 and C8–C13 phenyl rings, respectively. In the crystal packing (Fig. 3), molecules are stacked along the *c* axis by π···π interactions with  *Cg1*···*Cg2* = 3.7357 (12) Å: symmetry codes *x*, *y*, -1 + *z* and *x*, *y*, 1 + *z*;

### Experimental

The synthetic method has been described earlier (Al-Douh *et al.*, 2006, 2007). Single crystals suitable for *X*-ray diffraction were obtained by evaporation of a *n*-hexane solution at room temperature.

### Refinement

The H-atom attached to O1 is located from the difference Fourier map and refined as riding with the parent atom with an isotropic thermal parameter 1.2 times that of the parent atom. The methyl hydrogen atoms were fixed geometrically and refined using a rotating model with isotropic thermal parameters 1.5 that of the parent atom. The remaining hydrogen atoms were located in a difference map and refined freely with their isotropic thermal parameters 1.2 times those of the parent atoms.  The highest peak is located 0.96 Å from H2. The deepest hole is located 0.41 Å from H1O1.

### Crystal data

**Table:**

C_14_H_14_N_2_O_2_	*F*_000_ = 512
*M**_r_* = 242.27	*D*_x_ = 1.388 Mg m^−^^3^
Monoclinic, *P*2_1_/*c*	Mo *K*α radiation λ = 0.71073 Å
Hall symbol: -P 2ybc	Cell parameters from 4220 reflections
*a* = 13.2790 (6) Å	θ = 2.8–29.4º
*b* = 14.4810 (6) Å	µ = 0.09 mm^−^^1^
*c* = 6.1928 (3) Å	*T* = 100.0 (1) K
β = 103.116 (3)º	Needle, yellow
*V* = 1159.77 (9) Å^3^	0.45 × 0.15 × 0.05 mm
*Z* = 4	

### Data collection

**Table:**

Bruker SMART APEXII CCD area-detector diffractometer	3402 independent reflections
Radiation source: fine-focus sealed tube	2532 reflections with *I* > 2σ(*I*)
Monochromator: graphite	*R*_int_ = 0.052
*T* = 100.0(1) K	θ_max_ = 30.0º
φ and ω scans	θ_min_ = 2.1º
Absorption correction: multi-scan(SADABS; Bruker, 2005)	*h* = −18→18
*T*_min_ = 0.959, *T*_max_ = 0.996	*k* = −20→20
19968 measured reflections	*l* = −8→8

### Refinement

**Table:**

Refinement on *F*^2^	Secondary atom site location: difference Fourier map
Least-squares matrix: full	Hydrogen site location: inferred from neighbouring sites
*R*[*F*^2^ > 2σ(*F*^2^)] = 0.072	H atoms treated by a mixture of independent and constrained refinement
*wR*(*F*^2^) = 0.206	*w* = 1/[σ^2^(*F*_o_^2^) + (0.099*P*)^2^ + 1.0519*P*] where *P* = (*F*_o_^2^ + 2*F*_c_^2^)/3
*S* = 1.06	(Δ/σ)_max_ < 0.001
3402 reflections	Δρ_max_ = 0.66 e Å^−^^3^
194 parameters	Δρ_min_ = −0.31 e Å^−^^3^
Primary atom site location: structure-invariant direct methods	Extinction correction: none

### Special details

**Table:**

Experimental. The low-temperature data was collected with the Oxford Cyrosystem Cobra low-temperature attachment.
Geometry. All e.s.d.'s (except the e.s.d. in the dihedral angle between two l.s. planes) are estimated using the full covariance matrix. The cell e.s.d.'s are taken into account individually in the estimation of e.s.d.'s in distances, angles and torsion angles; correlations between e.s.d.'s in cell parameters are only used when they are defined by crystal symmetry. An approximate (isotropic) treatment of cell e.s.d.'s is used for estimating e.s.d.'s involving l.s. planes.
Refinement. Refinement of *F*^2^ against ALL reflections. The weighted *R*-factor *wR* and goodness of fit *S* are based on *F*^2^, conventional *R*-factors *R* are based on *F*, with *F* set to zero for negative *F*^2^. The threshold expression of *F*^2^ > σ(*F*^2^) is used only for calculating *R*-factors(gt) *etc*. and is not relevant to the choice of reflections for refinement. *R*-factors based on *F*^2^ are statistically about twice as large as those based on *F*, and *R*- factors based on ALL data will be even larger.

### Fractional atomic coordinates and isotropic or equivalent isotropic displacement parameters (Å^2^)

**Table:**

	*x*	*y*	*z*	*U*_iso_*/*U*_eq_	
O1	0.12067 (11)	1.03082 (11)	0.3256 (2)	0.0223 (4)	
H1O1	0.1550	1.0024	0.2388	0.027*	
O2	0.07156 (12)	1.12650 (11)	0.6488 (3)	0.0227 (4)	
N1	0.26011 (14)	0.97668 (12)	0.1182 (3)	0.0184 (4)	
N2	0.10257 (15)	0.90586 (15)	−0.2092 (3)	0.0251 (4)	
H1N2	0.058 (2)	0.909 (2)	−0.349 (5)	0.030*	
H2N2	0.110 (2)	0.954 (2)	−0.124 (5)	0.030*	
C1	0.20451 (16)	0.89186 (15)	−0.2205 (3)	0.0192 (4)	
C2	0.22641 (18)	0.83919 (16)	−0.3939 (4)	0.0225 (4)	
H2	0.165 (2)	0.8218 (19)	−0.497 (5)	0.027*	
C3	0.32708 (19)	0.82255 (14)	−0.4076 (4)	0.0219 (5)	
H3	0.339 (2)	0.7874 (19)	−0.528 (5)	0.026*	
C4	0.40918 (18)	0.85597 (15)	−0.2452 (4)	0.0216 (4)	
H4	0.477 (2)	0.8429 (19)	−0.253 (4)	0.026*	
C5	0.38917 (17)	0.90701 (14)	−0.0713 (4)	0.0196 (4)	
H5	0.447 (2)	0.9297 (18)	0.037 (5)	0.024*	
C6	0.28785 (16)	0.92618 (14)	−0.0560 (3)	0.0168 (4)	
C7	0.32762 (16)	1.02164 (14)	0.2604 (3)	0.0192 (4)	
H7	0.403 (2)	1.0240 (18)	0.247 (4)	0.023*	
C8	0.30011 (16)	1.07288 (14)	0.4405 (3)	0.0174 (4)	
C9	0.37772 (17)	1.11996 (14)	0.5926 (4)	0.0195 (4)	
H9	0.449 (2)	1.1215 (18)	0.569 (4)	0.023*	
C10	0.35346 (17)	1.16977 (14)	0.7632 (4)	0.0196 (4)	
H10	0.407 (2)	1.2018 (18)	0.862 (5)	0.024*	
C11	0.25165 (17)	1.17414 (14)	0.7875 (3)	0.0189 (4)	
H11	0.238 (2)	1.2072 (18)	0.902 (5)	0.023*	
C12	0.17424 (16)	1.12778 (14)	0.6423 (3)	0.0177 (4)	
C13	0.19759 (15)	1.07615 (14)	0.4659 (3)	0.0170 (4)	
C14	0.04456 (18)	1.17522 (17)	0.8283 (4)	0.0263 (5)	
H14A	0.0835	1.1510	0.9665	0.039*	
H14B	−0.0280	1.1678	0.8209	0.039*	
H14C	0.0600	1.2396	0.8181	0.039*	

### Atomic displacement parameters (Å^2^)

**Table:**

	*U*^11^	*U*^22^	*U*^33^	*U*^12^	*U*^13^	*U*^23^
O1	0.0173 (7)	0.0307 (8)	0.0180 (7)	−0.0022 (6)	0.0024 (6)	−0.0069 (6)
O2	0.0202 (8)	0.0294 (8)	0.0203 (7)	−0.0001 (6)	0.0081 (6)	−0.0067 (6)
N1	0.0189 (8)	0.0220 (9)	0.0144 (8)	0.0017 (7)	0.0042 (7)	0.0003 (6)
N2	0.0188 (9)	0.0345 (11)	0.0220 (10)	0.0020 (8)	0.0045 (8)	−0.0037 (8)
C1	0.0186 (10)	0.0229 (10)	0.0166 (9)	0.0026 (7)	0.0052 (8)	0.0020 (8)
C2	0.0253 (11)	0.0247 (10)	0.0156 (10)	−0.0014 (8)	0.0009 (8)	−0.0010 (8)
C3	0.0354 (12)	0.0175 (10)	0.0149 (9)	0.0024 (8)	0.0103 (9)	−0.0014 (7)
C4	0.0214 (10)	0.0217 (10)	0.0245 (11)	0.0023 (8)	0.0110 (9)	0.0031 (8)
C5	0.0182 (10)	0.0201 (9)	0.0196 (10)	−0.0015 (8)	0.0026 (8)	0.0005 (8)
C6	0.0219 (10)	0.0163 (9)	0.0126 (9)	0.0008 (7)	0.0045 (7)	0.0003 (7)
C7	0.0203 (10)	0.0207 (10)	0.0174 (10)	0.0007 (8)	0.0056 (8)	0.0022 (7)
C8	0.0200 (10)	0.0176 (9)	0.0154 (9)	0.0026 (7)	0.0054 (7)	0.0014 (7)
C9	0.0171 (10)	0.0220 (10)	0.0195 (10)	0.0003 (8)	0.0042 (8)	0.0032 (8)
C10	0.0205 (10)	0.0195 (9)	0.0168 (10)	−0.0020 (8)	0.0000 (8)	0.0002 (8)
C11	0.0258 (11)	0.0175 (9)	0.0139 (9)	0.0013 (8)	0.0055 (8)	−0.0014 (7)
C12	0.0165 (9)	0.0211 (9)	0.0161 (9)	0.0027 (7)	0.0049 (7)	0.0019 (7)
C13	0.0178 (10)	0.0188 (9)	0.0130 (9)	−0.0004 (7)	0.0003 (7)	0.0006 (7)
C14	0.0252 (11)	0.0362 (13)	0.0194 (11)	0.0023 (9)	0.0095 (9)	−0.0055 (9)

### Geometric parameters (Å, °)

**Table:**

O1—C13	1.351 (2)	C5—C6	1.398 (3)
O1—H1O1	0.8812	C5—H5	0.95 (3)
O2—C12	1.373 (2)	C7—C8	1.454 (3)
O2—C14	1.429 (3)	C7—H7	1.03 (3)
N1—C7	1.282 (3)	C8—C9	1.405 (3)
N1—C6	1.419 (3)	C8—C13	1.406 (3)
N2—C1	1.386 (3)	C9—C10	1.376 (3)
N2—H1N2	0.93 (3)	C9—H9	1.00 (3)
N2—H2N2	0.86 (3)	C10—C11	1.395 (3)
C1—C2	1.400 (3)	C10—H10	0.95 (3)
C1—C6	1.413 (3)	C11—C12	1.377 (3)
C2—C3	1.380 (3)	C11—H11	0.91 (3)
C2—H2	0.94 (3)	C12—C13	1.415 (3)
C3—C4	1.392 (3)	C14—H14A	0.9600
C3—H3	0.95 (3)	C14—H14B	0.9600
C4—C5	1.381 (3)	C14—H14C	0.9600
C4—H4	0.94 (3)		
			
C13—O1—H1O1	101.4	C8—C7—H7	117.8 (15)
C12—O2—C14	116.25 (17)	C9—C8—C13	119.34 (18)
C7—N1—C6	121.47 (18)	C9—C8—C7	119.24 (19)
C1—N2—H1N2	112.5 (18)	C13—C8—C7	121.42 (19)
C1—N2—H2N2	100 (2)	C10—C9—C8	120.3 (2)
H1N2—N2—H2N2	119 (3)	C10—C9—H9	119.9 (15)
N2—C1—C2	119.6 (2)	C8—C9—H9	119.5 (15)
N2—C1—C6	121.74 (19)	C9—C10—C11	120.5 (2)
C2—C1—C6	118.64 (19)	C9—C10—H10	118.3 (16)
C3—C2—C1	121.0 (2)	C11—C10—H10	121.2 (16)
C3—C2—H2	127.5 (17)	C12—C11—C10	120.37 (19)
C1—C2—H2	111.4 (17)	C12—C11—H11	121.2 (17)
C2—C3—C4	120.38 (19)	C10—C11—H11	118.4 (17)
C2—C3—H3	118.6 (16)	O2—C12—C11	125.89 (18)
C4—C3—H3	121.0 (17)	O2—C12—C13	114.12 (18)
C5—C4—C3	119.5 (2)	C11—C12—C13	119.99 (19)
C5—C4—H4	120.7 (17)	O1—C13—C8	121.42 (18)
C3—C4—H4	119.9 (17)	O1—C13—C12	119.14 (18)
C4—C5—C6	121.2 (2)	C8—C13—C12	119.44 (18)
C4—C5—H5	117.7 (17)	O2—C14—H14A	109.5
C6—C5—H5	121.1 (16)	O2—C14—H14B	109.5
C5—C6—C1	119.32 (18)	H14A—C14—H14B	109.5
C5—C6—N1	124.99 (19)	O2—C14—H14C	109.5
C1—C6—N1	115.67 (18)	H14A—C14—H14C	109.5
N1—C7—C8	121.86 (19)	H14B—C14—H14C	109.5
N1—C7—H7	120.3 (15)		
			
N2—C1—C2—C3	−178.8 (2)	C13—C8—C9—C10	−0.8 (3)
C6—C1—C2—C3	−1.4 (3)	C7—C8—C9—C10	179.32 (19)
C1—C2—C3—C4	1.7 (3)	C8—C9—C10—C11	0.0 (3)
C2—C3—C4—C5	−0.8 (3)	C9—C10—C11—C12	0.7 (3)
C3—C4—C5—C6	−0.4 (3)	C14—O2—C12—C11	−2.4 (3)
C4—C5—C6—C1	0.6 (3)	C14—O2—C12—C13	178.04 (18)
C4—C5—C6—N1	178.96 (19)	C10—C11—C12—O2	179.84 (19)
N2—C1—C6—C5	177.56 (19)	C10—C11—C12—C13	−0.7 (3)
C2—C1—C6—C5	0.2 (3)	C9—C8—C13—O1	−179.00 (18)
N2—C1—C6—N1	−0.9 (3)	C7—C8—C13—O1	0.9 (3)
C2—C1—C6—N1	−178.23 (19)	C9—C8—C13—C12	0.9 (3)
C7—N1—C6—C5	11.1 (3)	C7—C8—C13—C12	−179.28 (18)
C7—N1—C6—C1	−170.51 (19)	O2—C12—C13—O1	−0.7 (3)
C6—N1—C7—C8	−179.44 (18)	C11—C12—C13—O1	179.74 (18)
N1—C7—C8—C9	179.29 (19)	O2—C12—C13—C8	179.42 (17)
N1—C7—C8—C13	−0.6 (3)	C11—C12—C13—C8	−0.1 (3)

### Hydrogen-bond geometry (Å, °)

**Table:**

*D*—H···*A*	*D*—H	H···*A*	*D*···*A*	*D*—H···*A*
O1—H1O1···N1	0.88	1.77	2.602 (2)	158
N2—H1N2···O1^i^	0.93 (3)	2.56 (3)	3.030 (3)	111 (2)
N2—H1N2···O2^i^	0.93 (3)	2.29 (3)	3.181 (3)	161 (3)
N2—H2N2···N1	0.87 (3)	2.23 (3)	2.759 (3)	119 (2)
C3—H3···Cg1^ii^	0.94 (3)	2.64 (3)	3.458 (2)	145 (2)
C11—H11···Cg2^iii^	0.91 (3)	2.87 (3)	3.538 (2)	142 (2)

Symmetry codes: (i) −*x*, −*y*+2, −*z*; (ii) *x*, −*y*+1/2, *z*−3/2; (iii) *x*, −*y*+3/2, *z*−1/2.
